# Supplementary material for: The pitfalls of incidence-based time series regression for inferring the effects of weather on infectious diseases
Source: PLoS Comput Biol. 2026 Sep 10;22(9):e1014797. doi: 10.1371/journal.pcbi.1014797 (PMC13592718; doi:10.1371/journal.pcbi.1014797)
Supplement: Supplementary file 1 [file pcbi.1014797.s002.pdf]

Reviewer comments PCOMPBIOL-D-26-00663

This paper presents a simulation approach that explores how predictive estimates from time series regression (TSR) analysis of weather effects on disease incidence are subject to bias and inaccuracies. The authors use a simple simulation design to explore different potential causes of uncertainty and bias in these estimates, and conclude that the models are error prone even under conditions with low noise. These conclusions are then extrapolated to assume that the models will also perform poorly under more complex (i.e. real-world) conditions and that we need to be using more mechanistic approaches to infer weather effects from disease incidence data.

I think the general idea is both novel and interesting, however the manuscript could benefit from some clarifications and additional justification. I note that as a reviewer I am not particularly familiar with applications of TSR to environmental epidemiology; however, I am very familiar both with using this type of regression in other environmental and infectious disease modelling contexts and with mechanistic modelling. As it stands the paper has a reasonably narrow scope and intended audience, however many of the concepts discussed are relevant to a lot of predictive environmental modelling and a small amount of additional content could aid people in those fields in finding this topic useful as well. However, additional clarifications and nuance in discussion are required to achieve this.

My general comments on the manuscript are related to the following:

1. <u>Clarifications on TSR study design</u>
   a. A small amount of additional background information on TSR study design would be useful for a more general reader. E.g. explaining that it is a common method and that there are common TSR models used to determine relationships between weather and disease incidence.
   b. Additionally, the statement “The time-series regression (TSR) study design may be vulnerable to these issues. With this approach, the time series of incident cases is regressed against weather variables, with the resulting regression coefficients interpreted as causal effects” requires further information to support the statement that this method interprets relationships between weather variables and case incidence as causal effects, considering that regression is by design a correlative exercise. There are no references provided to support this statement, and I note that reference 9 (referred to later in the same paragraph), refers to associations between the time series and weather, not direct causation and that the review presented in reference 10 (mentioned in the same section) does not directly explore this. This may be a case of it being obvious to those familiar with TSR design and relate to my point above (i.e. more background information is required), however it would still be worthwhile to show that this is actually happening in practice.
2. <u>The role and meaning of causal modelling in further work.</u> The paper mostly differentiates between a regression approach where you're throwing everything at the wall and hoping something will stick and help you with prediction, vs mechanistic modelling with hypothesis driven relationships between variables. However, there is a third option, which is hypothesis driven regression. This is briefly touched on by the authors in the discussion (e.g. “More broadly, we propose that the choice of covariates in TSR models should be guided by causal models of the disease's natural history and other data-generating mechanisms, rather than by more general considerations (e.g.,

autocorrelation or seasonality [9,10]).”) however I think there is additional space to clarify that causal thinking can be included in TSR models and some examples of how this could work in practice. I.e. please clarify what “our results caution against the indiscriminate use of TSR models for estimating the effects of weather on disease dynamics” could mean in practice (for examples, drawing a DAG and providing hypotheses for how each covariate will influence disease incidence to justify its inclusion in the TSR model).

3. Weekly vs daily case incidence. Can the authors please provide justification for why weekly and not daily case incidence was used, considering that weather implicitly operates on shorter term timescales (i.e. daily) and it would seem logical to model it at this scale. It would also be interesting to add some discussion on what they would expect to see if finer-scale daily incidence data was used.
4. Acknowledgment of biological uncertainties in real-world applications and simplicity of the weather-incidence relationship used. The authors acknowledge that the simplified transmission model lacks real-world complexity, however do not currently provide enough justification for why the modelled comparison is analogous to a more complicated real-world example. Firstly, while the scenarios cover a number of different synthetic pathogens, the underlying relationship between the weather effects (a direct effect of relative humidity and temperature on the transmission rate) is consistent between all of them and a very simple causal relationship. One of the benefits of a correlative approach is that in a more complicated scenario, you don’t actually need to be able to quantify the mechanism, just have an idea of the suspected effect enough to justify including that variable in the model (and, a single variable may actually cover several correlated mechanisms in an umbrella way, with options to reduce variable complexity that are not available in a mechanistic framework). The authors have not justified why the problems identified in a simple scenario are relevant to the real world where dynamics are much messier and it would be substantially more difficult to use a mechanistic model instead of a correlative one. In the discussion the authors state that “accounting for these additional complexities would make the estimation problem more difficult and likely further reduce TSR performance” however do not currently provide evidence for why that would be the case. Surely accounting for additional complexities would also make fitting a mechanistic model more difficult? You also need a lot of information to parameterise a more complicated mechanistic model, information which is lacking for many disease (e.g. neglected tropical diseases). Surely a correlative approach is more likely to be used in this scenario. The authors have not compared a poorly parameterized, more complicated mechanistic model to a TSR design, so I do not think there is currently enough evidence to make the claim that “accounting for these additional complexities would make the estimation problem more difficult and likely further reduce TSR performance”.
5. I thank the authors for providing accessible code (GitFront was a new discovery for me). I note that the code has a detailed README and is structured with a renv file (so should be reproducible), however I have not run the code or examined the scripts in detail as part of this review.

Additional minor comments are provided below.

1. Note there are no line numbers which would help identify sections of text.

2. Section 3.5.5 TSR performance assessment – it would be useful to for the reader to include a statement that smaller values of RMSE and RMAB represent a more accurate estimate, either here or in the relevant figure captions.
3. Figure 2 – I was slightly confused that panel B representing the synthetic pathogens is not the same as Figure S2. Is it meant to be an illustrative example, in which case it doesn't matter that much, or is it meant to actually show a smaller version of Figure S2, in which case I note they are currently different.
4. Pg 14 - "In contrast, in realistic scenarios with latent variables affecting disease dynamics, TSR model estimates were substantially biased" – I am unsure what realistic scenarios the authors are referring to here? Given the examples are highly simplified.
5. Pg 15 "This is because in our model $C_t \approx I_t$ due to the fixed generation time, an equality that does not hold in more realistic transmission models with distributed generation times." I assume what the authors also mean is that this is an equality that does not hold in the real world either, and encourage them to change the language to directly reference that if so, rather than other hypothetical models.
