# Supplementary material for: The pitfalls of incidence-based time series regression for inferring the effects of weather on infectious diseases
Source: PLoS Comput Biol. 2026 Sep 10;22(9):e1014797. doi: 10.1371/journal.pcbi.1014797 (PMC13592718; doi:10.1371/journal.pcbi.1014797)
Supplement: Supplementary file 2 [file pcbi.1014797.s004.pdf]

**General key points**

We thank the editor and the two reviewers for their careful reading and thoughtful comments. We have thoroughly revised the text in response to these comments (including the results of a new sensitivity analysis)—please see our point-by-point response below.

1. *Please expand the background section to provide clearer context on time series regression models and how they are currently used in infectious disease modelling. This will help ensure the work is accessible to a broad readership.*

In response, we added a new paragraph to the introduction that provides context on the use of time series regression (TSR) models in infectious disease epidemiology and modeling.

2. *Please include a discussion of how causal inference approaches could inform the use of time series regression models in this context.*

In response, we clarified the text to highlight that our study—including the design of the TSR models—was framed within a causal inference framework. We also emphasize the following point, which may have been unclear in the initial manuscript: Our results suggest that, even when TSR models are formulated within a causal framework, they often fail at identifying weather effects. This is because the latent processes are dynamically complex for infectious diseases and hard to recapitulate with even flexible but non-mechanistic methods such as GAMs. In other words, while causal inference methods are useful to identify the causal problems associated with the TSR design, they cannot guarantee that estimates derived from common TSR model formulations will be causal.

3. *Please consider moderating the strength of your claims where appropriate and/or provide additional references and justification to support them.*

In response, we have revised the section on the limitations of our study in the discussion, in particular to clarify that further research into more causally elaborate formulations of TSR models is warranted. We also revised the abstract to tone down our statement advising against the indiscriminate use of TSR models.

**Reviewer 1**

***Major comments:***

*The introduction would benefit from additional discussion of why TSR approaches may be inappropriate in this setting, including more explicit reference to the assumptions these models rely on and why are they are violated in infectious disease dynamics. The use of TSR methods in infectious disease dynamics is nuanced and would benefit from addition intuition-building in the manuscript. In particular, incorporating a discussion of causal inference frameworks (e.g., DAGs) and how feedback mechanisms and temporal dependence complicate causal interpretation would strengthen the paper. I suggest at minimum including the following relevant papers*

- *Ackley SF, Mayeda ER, Worden L, Enanoria WTA, Glymour MM, Porco TC. Compartmental Model Diagrams as Causal Representations in Relation to DAGs. Epidemiol Methods. 2017;6(1):20060007. doi:10.1515/em-2016-0007*
- *Cobey S, Baskerville EB. Limits to Causal Inference with State-Space Reconstruction for Infectious Disease. PLOS ONE. 2016;11(12):e0169050. doi:10.1371/journal.pone.0169050*

We thank the reviewer for the suggested references, which we added to the main text (see the second and third paragraphs of the "*Introduction*"). Regarding TSR approaches, we developed the intuition for their limitations in the introduction, where we discussed measurement noise, data imperfections (e.g., due to under-reporting), and latent and dynamic variables, such as the size of the population susceptible to infection. In response to the reviewer, we have now expanded this part to better explain the potential problems with TSR approaches. We also agree with the reviewer's comment on the importance of causal inference frameworks (DAGs). However, we note that our entire study is framed within this causal framework. Specifically, we derived the DAG representing our transmission model (Figure 1) and used it throughout the text to explain the causal problems and motivate the different versions of the TSR models. Importantly, while DAGs are useful for clarifying causal problems, they do not guarantee that these problems can be solved in practice. For example, in our context, the DAG would indicate that covariates are needed to capture temporal variation in the susceptible population, but even a flexible method for estimating this covariate does not work well.

*The manuscript describes the framework as disease-agnostic; however, section 3.1.1 specifies a weather-dependent transmission relation based on work in respiratory viruses. If this assumption is central to transmission across all models - which it appears to be - it should be acknowledged when interpreting the results. Alternatively, the authors could provide justification for extending this relationship to other pathogens or consider exploring alternative transmission models.*

The reviewer raises an important point. Our simulations were based on a simple SIRS model, which generally works well for respiratory viruses and other respiratory pathogens. The weather-transmission relationship was motivated by earlier experimental work on how

temperature and relative humidity affect respiratory viruses, though we expect similar dependencies for other pathogens [1–6]. Because we repeated our simulations across various pathogen characteristics (mean age of infection and duration of immunity), our study can be considered disease-agnostic for pathogens that conform to this modeling framework. However, other diseases, e.g., vector-borne diseases, would require a different modeling framework, as they introduce additional complexities—such as transmission delays resulting from the intermediate vector—that would need to be incorporated. Though beyond the scope of this study, we view this as valuable future work, and we revised the text to discuss this limitation and perspective (see "*Discussion*", $9^{th}$ paragraph):

"*In particular, such lagged effects are especially relevant for vector-borne diseases, for which our modeling framework would need to be adapted accordingly.*"

*The poorer performance observed in the tropical location is interesting. This may related to nonlinear relationships between climate factors and transmission documents in tropical settings (e.g., Yuan H, Kramer SC, Lau EHY, Cowling BJ, Yang W. Modeling influenza seasonality in the tropics and subtropics. PLOS Computational Biology. 2021;17(6):e1009050. doi:10.1371/journal.pcbi.1009050 for influenza). Since the 4 locations were selected to represent different climate contexts, it would be more valuable to explicitly connect the differences in results to the underlying climates reflected in the study areas.*

The reviewer provides a valuable observation. In our simulations, we assumed the same functional form for the weather-transmission relationship across climates. Therefore, the observed performance differences reflect climate characteristics and how they translate into distinct epidemic patterns rather than any differences in the weather-transmission relationship. We note that poorer performance in the tropical climate primarily affected temperature effect estimates (Fig. 4, Table D in S1 appendix), which may be explained by the lower temperature variability in these settings (a point we added in the "*Results*" section, on page 15). However, we believe that a thorough understanding of how climate characteristics affect the identifiability of weather effects would require considering a wider range of climates. In response to the reviewer, we added a paragraph to the discussion to elaborate on these points (see "*Discussion*" section, $6^{th}$ paragraph):

"*Similarly, climatic characteristics affected TSR model performance, with less accurate estimation of temperature effects in the tropical climate considered. However, a more thorough understanding of how climatic characteristics affect the identifiability of weather effects would require consideration of a wider range of climates, which we see as valuable future work.*"

***Minor comments:***

- *In section 3.1, the term, "economical epidemic model" is unclear and should be defined or replaced and consistent with the legend in Figure 1 (which refers to it as causal)*

The term "economical epidemic model" was replaced with "parsimonious epidemic model". We note that in the legend in Figure 1, "causal" refers to the so-called "Causal directed acyclic graph" used to represent the causal links of the transmission model.

- *Please clarify the spatial resolution of the weather data and how it aligns with the selected locations*

We added the relevant information in section "*3.2.2 Data source*"

- *In section 3.3.1, the phrase, "capturing direct causal effects" may be overstated. While the model described is mechanistic and deterministic, additional justification is needed to classify it as causal.*

We replaced "capturing" with "quantifying" to avoid overstating the interpretation, and introduced additional causal justification (see "*3.3.1 Transmission rate & Weather effects"*):

"*Given the causal design (Fig.1), the parameters $\delta_{Te}$ and $\delta_{RH}$ quantify the direct causal effects of temperature and relative humidity on transmission rate.*"

## Reviewer 2

***Major comments:***

***Clarifications on TSR study design***

*A small amount of additional background information on TSR study design would be useful for a more general reader. E.g. explaining that it is a common method and that there are common TSR models used to determine relationships between weather and disease incidence.*

Thank you for this suggestion; in response, we added a paragraph on the TSR design to the introduction.

*Additionally, the statement "The time-series regression (TSR) study design may be vulnerable to these issues. With this approach, the time series of incident cases is regressed against weather variables, with the resulting regression coefficients interpreted as causal effects" requires further information to support the statement that this method interprets relationships between weather variables and case incidence as causal effects, considering that regression is by design a correlative exercise. There are no references provided to support this statement, and I note that reference 9 (referred to later in the same paragraph), refers to associations between the time series and weather, not direct causation and that the review presented in reference 10 (mentioned in the same section) does not directly explore this. This may be a case of it being obvious to those familiar with TSR design and relate to my point above (i.e. more background information is required), however it would still be worthwhile to show that this is actually happening in practice.*

The reviewer raises a critical point. While we agree that investigators using the TSR design often hedge their claims with vague terminology like "association," we argue that their aim is often causal rather than merely descriptive or predictive. In our previous perspective paper [7], we reviewed 33 studies using the TSR design and classified each study's objective as causal or predictive based on the presence of specific terms describing its aims or results (Table A in S1 appendix). Of the 33 studies, only four had an explicitly predictive objective, while the others addressed causality. More broadly, the use of associational language to mask causal aims is common in epidemiology [8]. We have included additional references and revised the text as follows to clarify these points (see second paragraph in the *"Introduction"*):

*"With this approach, the time series of observed incident cases is regressed against weather variables along with other covariates, and the resulting regression coefficients are often interpreted as estimates of the effect of weather on transmission or incidence"*

***The role and meaning of causal modelling in further work.*** *The paper mostly differentiates between a regression approach where you're throwing everything at the wall and hoping something will stick and help you with prediction, vs mechanistic modelling with hypothesis-driven relationships between variables. However, there is a third option, which is hypothesis-*

*driven regression. This is briefly touched on by the authors in the discussion (e.g. "More broadly, we propose that the choice of covariates in TSR models should be guided by causal models of the disease's natural history and other data-generating mechanisms, rather than by more general considerations (e.g., autocorrelation or seasonality [9,10])." ) however I think there is additional space to clarify that causal thinking can be included in TSR models and some examples of how this could work in practice. I.e. please clarify what "our results caution against the indiscriminate use of TSR models for estimating the effects of weather on disease dynamics" could mean in practice (for examples, drawing a DAG and providing hypotheses for how each covariate will influence disease incidence to justify its inclusion in the TSR model).*

We appreciate the reviewer's comment, but argue that our regression approach is hypothesis-driven. Indeed, our entire study is grounded in a causal framework, with an explicit DAG underlying our mechanistic simulation model (Figure 1). Similarly, as we explained in the methods section, our choice of covariates for the TSR models was hypothesis-driven and based on causal reasoning about the latent variables in this DAG. Our results suggest that, even when regression models are formulated within a causal framework, they often fail at identifying weather effects. This is because the latent processes underlying infectious diseases are dynamically complex and difficult to recapitulate with even flexible, yet non-mechanistic methods such as GAMs. More generally, we note that, even when the aim is causal, and models are designed within a causal framework, there is no guarantee that the estimates can be interpreted causally.

***Weekly vs daily case incidence.*** *Can the authors please provide justification for why weekly and not daily case incidence was used, considering that weather implicitly operates on shorter-term timescales (i.e. daily) and it would seem logical to model it at this scale. It would also be interesting to add some discussion on what they would expect to see if finer-scale daily incidence data were used.*

We agree that choosing a weekly time scale is an important modeling assumption. This choice—along with the assumption of a fixed generation time of 1 week—was motivated by two facts:

1. It allowed us to design a simple transmission model that could be represented exactly as a DAG.
2. It permitted the formulation of an "exact" TSR model equivalent to the transmission model (if the variables $S_t$ and $I_t$ were assumed known), which served as a control model for comparing the other TSR models (where the variables $S_t$ and $I_t$ were assumed unobserved, as would be the case in practice).

Altogether, this choice, while a simplifying assumption, provided an ideal experimental setting to test the TSR design. We note that our discrete-time transmission model iterates the epidemic dynamics from one week to the next, without considering values at intermediate time steps. Hence, weather values at shorter time scales would not affect the dynamics in this simulation setting.

That said, the reviewer raises a legitimate question about what would happen if the model instead operated in continuous time or on a shorter discrete time scale (with weather data entering as daily averages), while keeping the weekly time-scale aggregation for cases. This would create a mismatch between the exposure (weather variables) and the outcome (observed incidence rates), thereby posing an additional challenge for TSR. We view this as valuable future research; however, we argue that considering this issue would reinforce our message regarding the unreliability of TSR models. In response to the reviewer, we added the following text (see $10^{th}$ paragraph of *"Discussion"*) to discuss this point:

*"Third, we did not take into account the impact of the time scale used for data aggregation. Our discrete-time transmission model simulated the epidemic dynamics on a weekly basis, without considering values at intermediate time steps. In reality, however, the effects of weather occur over shorter time scales (for example, daily), which may not align with the aggregation of the data. This discrepancy could introduce further complexities, likely degrading the performance of estimation even more. At the same time, because surveillance epidemiological data are typically reported weekly, using weekly aggregation provides a more realistic setting for evaluating TSR models in practice."*

***Acknowledgement of biological uncertainties in real-world applications and simplicity of the weather-incidence relationship used.*** *The authors acknowledge that the simplified transmission model lacks real-world complexity, however do not currently provide enough justification for why the modelled comparison is analogous to a more complicated real-world example. Firstly, while the scenarios cover a number of different synthetic pathogens, the underlying relationship between the weather effects (a direct effect of relative humidity and temperature on the transmission rate) is consistent between all of them and a very simple causal relationship. One of the benefits of a correlative approach is that in a more complicated scenario, you don't actually need to be able to quantify the mechanism, just have an idea of the suspected effect enough to justify including that variable in the model (and, a single variable may actually cover several correlated mechanisms in an umbrella way, with options to reduce variable complexity that are not available in a mechanistic framework). The authors have not justified why the problems identified in a simple scenario are relevant to the real world where dynamics are much messier, and it would be substantially more difficult to use a mechanistic model instead of a correlative one. In the discussion the authors state that "accounting for these additional complexities would make the estimation problem more difficult and likely further reduce TSR performance" however do not currently provide evidence for why that would be the case. Surely accounting for additional complexities would also make fitting a mechanistic model more difficult? You also need a lot of information to parameterise a more complicated mechanistic model, information which is lacking for many diseases (e.g. neglected tropical diseases). Surely a correlative approach is more likely to be used in this scenario. The authors have not compared a poorly parameterised, more complicated mechanistic model to a TSR design, so I do not think there is currently enough evidence to make the claim that "accounting for these additional complexities would make the estimation problem more difficult and likely further reduce TSR performance".*

As noted in our reply to the previous reviewer's comment, we conducted a controlled numerical experiment using a deliberately simplified transmission model and simplifying assumptions to make the estimation problem as simple as possible for TSR models. The fact that TSR performs poorly in this context is striking, and we believe that our *a fortiori* argument that additional complexities would make things worse is justified.

That said, we appreciate the reviewer's comment that this statement needs additional justification. In response, we conducted a new analysis (the relevant code has been added to the GitFront repository) based on an updated transmission model with weather effects incorporated at two time lags (instead of just one in the initial analysis). The corresponding methods are now reported in the revised manuscript (see Section A in the S1 appendix), and the results are summarized in the new Figure L in the S1 appendix, reproduced below for the reviewer's convenience. We find that, compared with the initial analysis, TSR performance degrades, with higher RMAB and RMSE for each weather variable's two lagged effects. Hence, this new analysis provides preliminary support for our claim.

Regarding the reviewer's points on mechanistic models, we agree that parametrizing them can be difficult and requires substantial amounts of data. Although our study focuses on TSR models, we note that recent advances in statistical inference methods for complex dynamic transmission models (e.g., partially observed Markov approaches [9,10]) make such parametrization increasingly feasible. The growing number of applications across diseases (such as influenza, RSV, and COVID-19 [11,12]) attests that weather effects can be estimated using rigorous mechanistic transmission models rather than TSR models. That said, we agree that these methods are more challenging to apply than TSR models, and we view further research to improve TSR design as valuable. We revised the manuscript to elaborate on this point (Discussion):

"*More generally, more causally elaborate formulations may improve TSR models and, given the substantial amounts of data needed for reliable parametrization of transmission models, further research on the TSR design is worthwhile.*"

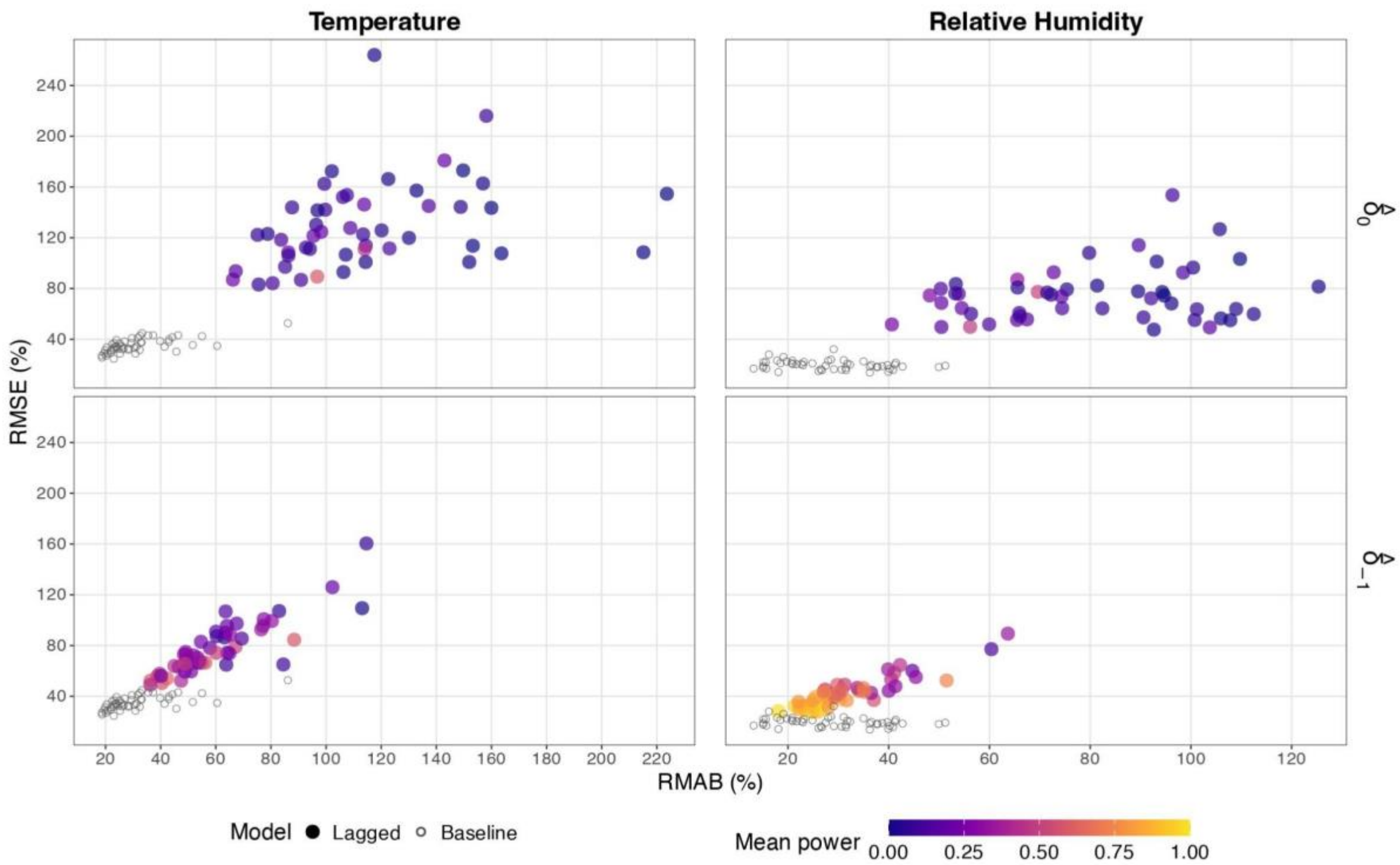


**Fig. L: Assessment of TSR model performance with additional lagged effect in Toronto.** Each point represents a tested synthetic pathogen, with TSR performance quantified by the Relative Mean Absolute Bias (RMAB; x-axis, averaged across replicates) and the Relative Mean Standard Error (RMSE; y-axis, averaged across replicates). The color scale represents the scenario-averaged power, defined as the probability that the model correctly recovers the sign of the true effect. For comparison, the grey points indicate the performance of the baseline test model without lagged weather effects (the weather effect estimated for the baseline is repeated across the two rows). Columns correspond to the two weather variables. Rows distinguish between the two lagged weather effects.

With the new transmission rate defined as:

$$\log \beta_t = \log \bar{\beta} + \delta_{Te,0} Te_t{}' + \delta_{Te,-1} Te'_{t-1} + \delta_{RH,0} RH'{}_t + \delta_{RH,-1} RH'_{t-1}$$

***Minor comments:***

- *Note that there are no line numbers which would help identify sections of text.*

Line numbers have now been added for the main text and supplementary material.

- *Section 3.5.5 TSR performance assessment – it would be useful to for the reader to include a statement that smaller values of RMSE and RMAB represent a more accurate estimate, either here or in the relevant figure captions.*

  We added a brief explanation in the main text after defining the measures (see section *"3.5.3 TSR performance assessment"*)

- *Figure 2 – I was slightly confused that panel B, representing the synthetic pathogens is not the same as Figure S2. Is it meant to be an illustrative example, in which case it doesn't matter that much, or is it meant to actually show a smaller version of Figure S2, in which case I note they are currently different.*

  Panel B in Fig.2, which was indeed an illustrative example, has been replaced and is now consistent with Figure B in S1 appendix.

- *Pg 14 - "In contrast, in realistic scenarios with latent variables affecting disease dynamics, TSR model estimates were substantially biased" – I am unsure what realistic scenarios the authors are referring to here? Given that the examples are highly simplified.*

  We rephrased and expanded the sentence to explicitly refer to the performance observed during the sensitivity analysis, in which additional realistic sources of complexity were incorporated into the system.

- *Pg 15 "This is because in our model $C_t \approx I_t$ due to the fixed generation time, an equality that does not hold in more realistic transmission models with distributed generation times." I assume what the authors also mean is that this is an equality that does not hold in the real world either, and encourage them to change the language*

  The sentence has been changed to:

  *"This is because in our model $C_t \approx I_t$ , due to the fixed generation time, a property that does not hold in the real world, due to distributed generation times."*

## References


1. Jong JG. The survival of measles virus in air, in relation to the epidemiology of measles. Arch Virol. 1965;16: 97–102.

2. Ehrlich R, Miller S, Walker RL. Relationship between atmospheric temperature and survival of airborne bacteria. Appl Microbiol. 1970;19: 245–249.

3. Ehrlich R, Miller S. Survival of airborne Pasteurella tularensis at different atmospheric

temperatures. Appl Microbiol. 1973;25: 369–372.

4. Wathes CM, Howard K, Webster AJ. The survival of Escherichia coli in an aerosol at air temperatures of 15 and 30 degrees C and a range of humidities. J Hyg (Lond). 1986;97: 489–496.

5. Theunissen HJ, Lemmens-den Toom NA, Burggraaf A, Stolz E, Michel MF. Influence of temperature and relative humidity on the survival of Chlamydia pneumoniae in aerosols. Appl Environ Microbiol. 1993;59: 2589–2593.

6. Wright DN, Bailey GD, Goldberg LJ. Effect of temperature on survival of airborne Mycoplasma pneumoniae. J Bacteriol. 1969;99: 491–495.

7. Barrero Guevara LA, Kramer SC, Kurth T, Domenech de Cellès M. Causal inference concepts can guide research into the effects of climate on infectious diseases. Nat Ecol Evol. 2025;9: 349–363.

8. Labrecque JA, Kezios KL. How and when to use causal and associational language. BMJ. 2026;392: e085749.

9. King AA, Nguyen D, Ionides EL. Statistical inference for partially observed Markov processes via theRPackagepomp. J Stat Softw. 2016;69: 1–43.

10. Ionides EL, Nguyen D, Atchadé Y, Stoev S, King AA. Inference for dynamic and latent variable models via iterated, perturbed Bayes maps. Proc Natl Acad Sci U S A. 2015;112: 719–724.

11. Du X, King AA, Woods RJ, Pascual M. Evolution-informed forecasting of seasonal influenza A (H3N2). Sci Transl Med. 2017;9: eaan5325.

12. Drake JM, Handel A, Marty É, O'Dea EB, O'Sullivan T, Righi G, et al. A data-driven semi-parametric model of SARS-CoV-2 transmission in the United States. PLoS Comput Biol. 2023;19: e1011610.
