# Supplementary material for: The pitfalls of incidence-based time series regression for inferring the effects of weather on infectious diseases
Source: PLoS Comput Biol. 2026 Sep 10;22(9):e1014797. doi: 10.1371/journal.pcbi.1014797 (PMC13592718; doi:10.1371/journal.pcbi.1014797)
Supplement: Peer review history

# Peer review: Decision Letter 0

**DOI:** 10.1371/journal.pcbi.1014797.r001

**Authors:** Jennifer Flegg, Nicholas Geard

10 Jun 2026

The pitfalls of incidence-based time series regression for inferring the effects of weather on infectious diseases

PLOS Computational Biology

Dear Dr. Gemo,

Thank you for submitting your manuscript to PLOS Computational Biology. After careful consideration, we feel that it has merit but does not fully meet PLOS Computational Biology's publication criteria as it currently stands. Therefore, we invite you to submit a revised version of the manuscript that addresses the points raised during the review process.

Please submit your revised manuscript by Aug 10 2026 11:59PM. If you will need more time than this to complete your revisions, please reply to this message or contact the journal office at ploscompbiol@plos.org. When you're ready to submit your revision, log on to https://www.editorialmanager.com/pcompbiol/ and select the 'Submissions Needing Revision' folder to locate your manuscript file.

Please include the following items when submitting your revised manuscript:

* A letter that responds to each point raised by the editor and reviewer(s). You should upload this letter as a separate file labeled 'Response to Reviewers'. This file does not need to include responses to formatting updates and technical items listed in the 'Journal Requirements' section below.

* A marked-up copy of your manuscript that highlights changes made to the original version. You should upload this as a separate file labeled 'Revised Manuscript with Track Changes'.

* An unmarked version of your revised paper without tracked changes. You should upload this as a separate file labeled 'Manuscript'.

We look forward to receiving your revised manuscript.

Kind regards,

Nicholas Geard

Academic Editor

PLOS Computational Biology

Jennifer Flegg

Section Editor

PLOS Computational Biology

**Additional Editor Comments (if provided):**

Thank you for your submission. The reviewers agree that your manuscript addresses a novel and important topic and has potential to be of interest to the PLOS Computational Biology readership. However, they identified several areas where revisions are needed to improve the manuscript's accessibility, clarity and impact. In particular, we ask that you address the following key points:

1. Please expand the background section to provide clearer context on time series regression models and how they are currently used in infectious disease modelling. This will help ensure the work is accessible to a broad readership.

2. Please include a discussion of how causal inference approaches could inform the use of time series regression models in this context.

3. Please consider moderating the strength of your claims where appropriate and/or provide additional references and justification to support them.

**Journal Requirements:**

1) Please ensure that the CRediT author contributions listed for every co-author are completed accurately and in full.

At this stage, the following Authors/Authors require contributions: Pietro Gemo, Laura Andrea Barrero Guevara, Cana Kussmaul, Sarah C. Kramer, and Matthieu Domenech de Cellès. Please ensure that the full contributions of each author are acknowledged in the "Add/Edit/Remove Authors" section of our submission form.

The list of CRediT author contributions may be found here: https://journals.plos.org/ploscompbiol/s/authorship#loc-author-contributions

2) We ask that a manuscript source file is provided at Revision. Please upload your manuscript file as a .doc, .docx, .rtf or .tex. If you are providing a .tex file, please upload it under the item type u2018LaTeX Source Fileu2019 and leave your .pdf version as the item type u2018Manuscriptu2019.

3) We have noticed that you have uploaded Supporting Information files, but you have not included a list of legends. Please add a full list of legends for your Supporting Information files after the references list.

4) In the online submission form, you indicated that your data will be submitted to a repository upon acceptance. We strongly recommend all authors deposit their data before acceptance, as the process can be lengthy and hold up publication timelines. Please note that, though access restrictions are acceptable now, your entire minimal dataset will need to be made freely accessible if your manuscript is accepted for publication. This policy applies to all data except where public deposition would breach compliance with the protocol approved by your research ethics board. If you are unable to adhere to our open data policy, please kindly revise your statement to explain your reasoning and we will seek the editor's input on an exemption.

5) Please amend your detailed Financial Disclosure statement. This is published with the article. It must therefore be completed in full sentences and contain the exact wording you wish to be published.

1) State the initials, alongside each funding source, of each author to receive each grant. For example: "This work was supported by the National Institutes of Health (####### to AM; ###### to CJ) and the National Science Foundation (###### to AM)."

2) State what role the funders took in the study. If the funders had no role in your study, please state: "The funders had no role in study design, data collection and analysis, decision to publish, or preparation of the manuscript."

3) If any authors received a salary from any of your funders, please state which authors and which funders.

6) We have amended your Competing Interest statement to comply with journal style. We kindly ask that you double check the statement and let us know if anything is incorrect.

**Reviewers' comments:**

Reviewer's Responses to Questions

**Comments to the Authors:**

**Please note here if the review is uploaded as an attachment.**

Reviewer #1: Summary: This paper addresses an important topic in understanding the dynamics of seasonal outbreaks, particularly when incorporating the influences of weather, which also exhibit strong seasonal patterns. The simulation framework, where the authors can specify the true effects of climate on transmission and then assess whether time-series regression (TSR) can recover the modeled effects, is a strong approach. They find that TSR methods struggle to recover true weather effects on “observed” (albeit simulated) outbreaks, particularly depending on the climate variable or amount of measurement noise.

Major comments:

- The introduction would benefit from additional discussion of why TSR approaches may be inappropriate in this setting, including more explicit reference to the assumptions these models rely on and why are they are violated in infectious disease dynamics. The use of TSR methods in infectious disease dynamics is nuanced and would benefit from addition intuition-building in the manuscript. In particular, incorporating a discussion of causal inference frameworks (e.g., DAGs) and how feedback mechanisms and temporal dependence complicate causal interpretation would strengthen the paper. I suggest at minimum including the following relevant papers

- Ackley SF, Mayeda ER, Worden L, Enanoria WTA, Glymour MM, Porco TC. Compartmental Model Diagrams as Causal Representations in Relation to DAGs. Epidemiol Methods. 2017;6(1):20060007. doi:10.1515/em-2016-0007

- Cobey S, Baskerville EB. Limits to Causal Inference with State-Space Reconstruction for Infectious Disease. PLOS ONE. 2016;11(12):e0169050. doi:10.1371/journal.pone.0169050

- The manuscript describes the framework as disease-agnostic; however, section 3.1.1 specifies a weather-dependent transmission relation based on work in respiratory viruses. If this assumption is central to transmission across all models - which it appears to be - it should be acknowledged when interpreting the results. Alternatively, the authors could provide justification for extending this relationship to other pathogens or consider exploring alternative transmission models

- The poorer performance observed in the tropical location is interesting. This may related to nonlinear relationships between climate factors and transmission documents in tropical settings (e.g., Yuan H, Kramer SC, Lau EHY, Cowling BJ, Yang W. Modeling influenza seasonality in the tropics and subtropics. PLOS Computational Biology. 2021;17(6):e1009050. doi:10.1371/journal.pcbi.1009050 for influenza). Since the 4 locations were selected to represent different climate contexts, it would be more valuable to explicitly connect the differences in results to the underlying climates reflected in the study areas

Minor comments:

- In section 3.1, the term, “economical epidemic model” is unclear and should be defined or replaced and consistent with the legend in Figure 1 (which refers to it as causal)

- Please clarify the spatial resolution of the weather data and how it aligns with the selected locations

- In section 3.3.1, the phrase, “capturing direct causal effects” may be overstated. While the model described is mechanistic and deterministic, additional justification is needed to classify it as causal

Reviewer #2: Please see attached

**********

**Have the authors made all data and (if applicable) computational code underlying the findings in their manuscript fully available?**

The PLOS Data policy requires authors to make all data and code underlying the findings described in their manuscript fully available without restriction, with rare exception (please refer to the Data Availability Statement in the manuscript PDF file). The data and code should be provided as part of the manuscript or its supporting information, or deposited to a public repository. For example, in addition to summary statistics, the data points behind means, medians and variance measures should be available. If there are restrictions on publicly sharing data or code —e.g. participant privacy or use of data from a third party—those must be specified.

Reviewer #1: Yes

Reviewer #2: Yes

**********

PLOS authors have the option to publish the peer review history of their article (what does this mean?). If published, this will include your full peer review and any attached files.

If you choose “no”, your identity will remain anonymous but your review may still be made public.

**Do you want your identity to be public for this peer review?**  For information about this choice, including consent withdrawal, please see our Privacy Policy.

Reviewer #1: No

Reviewer #2: No

[NOTE: If reviewer comments were submitted as an attachment file, they will be attached to this email and accessible via the submission site. Please log into your account, locate the manuscript record, and check for the action link "View Attachments". If this link does not appear, there are no attachment files.]

**Figure resubmission:**

**Reproducibility:**

?>

Attachment Submitted filename: PCOMPBIOL-D-26-00663-reviewer-response.pdf

# Author comment: Author response to Decision Letter 1

**DOI:** 10.1371/journal.pcbi.1014797.r002

17 Aug 2026

Attachment Submitted filename: Response_to_Reviewers.pdf

# Peer review: Decision Letter 1

**DOI:** 10.1371/journal.pcbi.1014797.r003

**Authors:** Jennifer Flegg, Nicholas Geard

1 Sep 2026

Dear PhD Candidate Gemo,

We are pleased to inform you that your manuscript 'The pitfalls of incidence-based time series regression for inferring the effects of weather on infectious diseases' has been provisionally accepted for publication in PLOS Computational Biology.

Before your manuscript can be formally accepted you will need to complete some formatting changes, which you will receive in a follow up email. A member of our team will be in touch with a set of requests.

Please note that your manuscript will not be scheduled for publication until you have made the required changes, so a swift response is appreciated.

IMPORTANT: The editorial review process is now complete. PLOS will only permit corrections to spelling, formatting or significant scientific errors from this point onwards. Requests for major changes, or any which affect the scientific understanding of your work, will cause delays to the publication date of your manuscript.

Should you, your institution's press office or the journal office choose to press release your paper, you will automatically be opted out of early publication. We ask that you notify us now if you or your institution is planning to press release the article. All press must be co-ordinated with PLOS.

Thank you again for supporting Open Access publishing; we are looking forward to publishing your work in PLOS Computational Biology.

Best regards,

Nicholas Geard

Academic Editor

PLOS Computational Biology

Jennifer Flegg

Section Editor

PLOS Computational Biology

***********************************************************

Reviewer's Responses to Questions

**Comments to the Authors:**

**Please note here if the review is uploaded as an attachment.**

Reviewer #2: I thank the authors for their thorough consideration of reviewer and editor comments and subsequent revisions to the manuscript. The resubmission is much improved in terms of readability and accessibility for a more general audience, and in terms of the authors justifying their study design and/or discussing the limitations. I appreciate the addition of the sensitivity analysis as well.

I believe the authors have addressed all of my major concerns in a considered and comprehensive manner. As a suggestion, if the authors wanted to add some subheadings to the now quite long Discussion section it would help improve readability, however that is merely a suggestion and entirely optional on their part. I have no additional changes to suggest.

**********

**Have the authors made all data and (if applicable) computational code underlying the findings in their manuscript fully available?**

The PLOS Data policy requires authors to make all data and code underlying the findings described in their manuscript fully available without restriction, with rare exception (please refer to the Data Availability Statement in the manuscript PDF file). The data and code should be provided as part of the manuscript or its supporting information, or deposited to a public repository. For example, in addition to summary statistics, the data points behind means, medians and variance measures should be available. If there are restrictions on publicly sharing data or code —e.g. participant privacy or use of data from a third party—those must be specified.

Reviewer #2: Yes

**********

PLOS authors have the option to publish the peer review history of their article (what does this mean?). If published, this will include your full peer review and any attached files.

If you choose “no”, your identity will remain anonymous but your review may still be made public.

**Do you want your identity to be public for this peer review?**  For information about this choice, including consent withdrawal, please see our Privacy Policy.

Reviewer #2: No

# Editor report: Acceptance letter

**DOI:** 10.1371/journal.pcbi.1014797.r004

**Authors:** Jennifer Flegg, Nicholas Geard

PCOMPBIOL-D-26-00663R1

The pitfalls of incidence-based time series regression for inferring the effects of weather on infectious diseases

Dear Dr Gemo,

I am pleased to inform you that your manuscript has been formally accepted for publication in PLOS Computational Biology. Your manuscript is now with our production department and you will be notified of the publication date in due course.

The corresponding author will soon be receiving a typeset proof for review, to ensure errors have not been introduced during production. Please review the PDF proof of your manuscript carefully, as this is the last chance to correct any errors. Please note that major changes, or those which affect the scientific understanding of the work, will likely cause delays to the publication date of your manuscript.

Soon after your final files are uploaded, unless you have opted out, the early version of your manuscript will be published online. The date of the early version will be your article's publication date. The final article will be published to the same URL, and all versions of the paper will be accessible to readers.

For Research, Software, and Methods articles, you will receive an invoice from PLOS for your publication fee after your manuscript has reached the completed accept phase. If you receive an email requesting payment before acceptance or for any other service, this may be a phishing scheme. Learn how to identify phishing emails and protect your accounts at https://explore.plos.org/phishing.

Thank you again for supporting PLOS Computational Biology and open-access publishing. We are looking forward to publishing your work!

With kind regards,

Sharmila Kamatchi

PLOS Computational Biology | Carlyle House, Carlyle Road, Cambridge CB4 3DN | United Kingdom ploscompbiol@plos.org | Phone +44 (0) 1223-442824 | ploscompbiol.org | @PLOSCompBiol
